# Beyond Gender: Interoceptive Sensibility as a Key Predictor of Body Image Disturbances

**DOI:** 10.3390/bs14010025

**Published:** 2023-12-28

**Authors:** Akansha M. Naraindas, Marina Moreno, Sarah M. Cooney

**Affiliations:** School of Psychology, University College Dublin, D04 F6X4 Dublin, Ireland; marinamorenocorreo@gmail.com

**Keywords:** body image, body schema, gender, interoceptive sensibility, motor imagery

## Abstract

Body image disturbance (BID) involves negative attitudes towards shape and weight and is associated with lower levels of interoceptive sensibility (IS) (the subjective perceptions of internal bodily states). This association is considered a risk factor for developing eating disorders (EDs) and is linked to altered sensorimotor representations of the body (i.e., body schema). BIDs manifest across genders and are currently understudied in men. This study investigated gender-related differences in BID and its relationship to the body schema and IS. Data were collected from 86 men and 86 women. BID was assessed using questionnaires measuring self-objectification, state, and trait body dissatisfaction. IS was measured via the MAIA-2. The body schema was indexed via an embodied mental rotation task. Results showed that women reported higher BID than men across all scales. Gender differences in sub-components of interoceptive sensibility were found. Overall, both gender and interoceptive sensibility predicted BID. However, interoceptive sensibility exhibited its own unique association with BID beyond the influence of gender. BID, IS and gender were not significant predictors of performance in the body schema task. Therefore, while gender predicts differences in BID and interoceptive sensibility, there was no evidence of gender-related differences in body schema.

## 1. Introduction

Body image is a complex construct that involves a person’s feelings and attitudes towards their size, shape, and weight [1]. Negative body image is a growing public health concern and is closely tied to significant mental health consequences worldwide, including depression, anxiety, low self-esteem, and disordered eating [2]. Factors such as gender identity play an important role in shaping an individual’s experience of their body. Research suggests that women tend to report higher levels of negative body image (e.g., body dissatisfaction) and consequently, higher rates of eating disorders (EDs) than men across different age groups [3,4,5,6,7]. However, other research suggests that men are becoming increasingly dissatisfied with their bodies, and this discontent is linked to disordered eating patterns, compulsive exercise behaviours, and the use of steroids [8,9,10,11]. Body image disturbances (BIDs) involve the distortion of perception, behaviour, or cognition related to weight or shape, and are a recognised risk factor in the onset of eating disorders [12]. Research indicates that BIDs in women are associated with atypical internal sensory experiences, including lower levels of awareness of and attention towards internal physiological states i.e., interoception (see Badoud and Tsakiris, 2017 [13] for review). BIDs are also associated with disturbances in the body schema—an implicit sensorimotor representation of the body for action [14]. To date links between BID and body schema have primarily been researched in the context of young women diagnosed with EDs [15,16,17,18,19]. Less than 1% of all research on BIDs has looked at presentations in men, posing a significant challenge in discerning the applicability of clinical and empirical findings that can be drawn about male body image from studies primarily focused on women with EDs [20]. Therefore, this study examines, for the first time, gender-related differences in BID and its relationship to interoception and the body schema.

Men and women experience different socio-cultural pressures and expectations related to body image [21]. While women are faced with heightened thin-ideal appearance pressures [22], men’s body image pressures are often associated with a drive for muscularity [8]. However, this is an oversimplified view of body image, as body ideals have changed rapidly over the past decade with all genders converging on body ideals and demonstrating both a drive for muscularity [23], aversiveness to body fat [24] and a drive for thinness [25,26]. Research indicates that women tend to demonstrate more critical appearance-related comparisons [27] and higher levels of body dissatisfaction compared to men [3,28,29]. However, body dissatisfaction is complex and can either be experienced as an immediate state or a stable trait over time [30]. Currently, most body image research focuses on long-term trait tendencies regarding weight and shape evaluation, with less attention paid to current states of negative body image [31,32]. Studies show that both trait and state body dissatisfaction independently predict eating pathology in women [33], and state body dissatisfaction has a unique contribution to altered eating patterns, even beyond trait concerns [34]. Nevertheless, the relationship between trait and state body dissatisfaction remains unclear, particularly in terms of gender differences and their potential shared consequences across genders.

Body dissatisfaction has been linked to self-objectification in both men and women [35]. The objectification theory was initially formulated to explain negative body image in women within a culture that sexually objectifies women’s bodies [36,37]. However, the increased prevalence of muscle-building practices and the sexualized portrayals of men’s bodies in the media have recently also contributed to elevated levels of self-objectification among men [38,39,40]. Self-objectification is characterised by two distinct facets: body surveillance, which involves the regular scrutiny of one’s physical appearance, and body shame, stemming from not meeting culturally internalized beauty ideals [36]. In women, elevated self-objectification, and BIDs in general, are believed to lead to an external focus on their physical attributes, particularly their appearance [41]. This tendency is said to result in a diminished awareness of their internal bodily functions (e.g., interoception) [13] as well as alterations in other aspects of body representations such as the body schema [41,42]. However, the evidence regarding the connections between BID, interoception, and the body schema in men is equivocal, raising questions about whether these findings are specific to women.

Interoception is essential for body representation, involving the awareness of afferent signals that convey the body’s inner physiological state [43]. There are gender differences in how individuals perceive and respond to their internal physiological states and how they use these signals for higher-order cognitive processes [44]. Women typically demonstrate reduced interoceptive sensitivity in tasks that measure heartbeat detection [45,46] but greater sensitivity to gastric signalling than men [44]. There is an established link between negative body image and reduced interoceptive processing in women (body image and interoceptive awareness: [47,48], body image and interoceptive accuracy: [49,50,51] body image and interoceptive sensibility: [52,53]). However, comparably less is known about this association in men. One area of interoception that has gained attention in body image research is interoceptive sensibility (IS), i.e., the subjective perception and awareness of bodily signals [54]. IS is typically assessed using the 32-item multidimensional assessment of interoceptive awareness (MAIA), which assesses eight different factors related to interoceptive awareness [55]. Studies show that women typically report higher levels of emotional awareness in the MAIA-2 compared to men (this subscale assesses the awareness of the relationship between emotional and bodily states) [56,57]. Conversely, men tend to report higher levels of body trust, self-regulation, and attention regulation across their respective sub-scales compared to women [45,57]. Reduced interoceptive sensibility is frequently linked with increased negative body image in women [57,58,59,60,61]. However, research examining the connection between IS and BIDs in men is limited, although some nascent evidence is beginning to emerge. For example, a recent study found significant negative relationships between the *Not distracting*, *Not worrying*, and *Noticing* sub-scales of the MAIA-2 and scores on the muscle dysmorphic disorder inventory [62]. Additionally, in Todd et al.’s (2019) study on gender, IS, and body image, the MAIA-2 sub-scales were found to predict positive body appreciation, functionality, and pride, beyond the influence of gender [57]. However, IS had a relatively weaker association with negative body image factors, such as preoccupation with weight and appearance orientation [57]. As such, the current understanding of the relationship between IS and different aspects of negative body image in men remains uncertain.

Furthermore, BIDs in women have also been associated with disturbances in the body schema. Experimental studies demonstrate that women with EDs tend to perceive their bodies as larger than their true size and engage with their bodies as if they occupy a greater space during action [15,16]. To date, body schema distortions linked to BIDs have not been established in men conclusively, with a few studies indicating that men generally tend to underestimate their body size in body size estimation tasks [63,64]. However, the body schema is an action-based representation encompassing both physical actions [14,65] and mental simulation of movements i.e., motor imagery [66]. Motor imagery is usually assessed using body-based mental rotation paradigms such as the own body transformation task (OBT) [67]. In the OBT, individuals are instructed to mentally simulate movements or rotate specific body parts to match their physical positions. This transformation, known as an egocentric mental transformation [68,69], is based on the internal representation of one’s body schema. Women with BIDs demonstrate reduced accuracy and slower reaction times than healthy controls in motor imagery tasks that involve mentally rotating their bodies, compared to rotating objects [19,70,71]. However, as men are considered to perform better at mental rotation tasks than women [72,73,74], it is unclear whether BID would affect their ability to use their body schema to make egocentric mental rotations in the OBT task.

An explanation for modified egocentric mental rotations in individuals with BIDs comes from the allocentric lock theory (ALT) [42]. The ALT suggests that BIDs hinder egocentric processing, leading to the inability to update the allocentric mental representation of the body schema, resulting in a distorted body representation. This occurs due to exogenous stressors like negative body image (e.g., body dissatisfaction, body objectification) that influence how internal body-related sensory information (e.g., interoception) is processed [75]. The ALT, originally formulated to account for body schema disturbances among women, has not yet been applied to men, giving rise to uncertainties about the relationship between BID, egocentric processing, and interoception in men. Hence, the present study closely follows the methodology of Naraindas and Cooney (2023), where we examined the relationship between BID and IS throughout the course of female adulthood [53]. We will apply the same methodology to investigate differences in BID and IS between men and women and investigate the role of gender in the relationship between BID, IS, and body schema.

Overall, we hypothesise that:

1. Women will have higher levels of BID than males across all scales [3].
2. We also expect that there will be a difference in MAIA-2 sub-scale scores between men and women [45,57] with men having higher total scores than women.
3. We expect that men will outperform women in the OBT task [72].
4. We expect a relationship between BID and body schema as indexed by performance in the OBT task across gender [53].

## 2. Materials and Methods

This was an online cross-sectional study that employed a within-subjects repeated measures design. The study was presented online on Qualtrics [76] and Psychopy (version 2021.1.3; [77]) via the Pavlovia platform (https://pavlovia.org/, accessed on 19 January 2022). The analysis plan was pre-registered on aspredicted.org (accessed on 13 April 2023) (AsPredicted #128634).

### 2.1. Participants

A total of 170 participants, 86 men (M = 20.5, SD = 1.9, Range = 18–24) and 86 women (M = 21.3, SD = 1.5, Range = 18–25), took part in this study. Men were recruited online, and women were selected from a larger cross-sectional data set from a study with the same methods conducted by the authors [53]. The inclusion criteria were as follows: all participants were right-handed or ambidextrous, possessed normal or corrected-to-normal vision, had no neurological conditions, and did not self-report any history of eating disorders. The study was approved by the Human Research Ethics Committee at University College Dublin in line with the declaration of Helsinki and all the participants gave informed consent to take part.

Women from the broader data set were matched to men by age (18–25 age group) and ethnicity. Specifically, they were selected from the five most frequently occurring EU member countries in the large dataset: the UK (N = 20), Italy (N = 25), Greece (N = 9), Portugal (N = 14), and Poland (N = 18), to match with the Irish males recruited for the study. All the selected countries have a human development index score of ≥0.850 [78], indicating similar levels of high socioeconomic development. As body image concerns are believed to be associated with westernization, urbanization, and economic growth [79,80,81] we expect the selected countries to present roughly similar body image concerns to each other and a matched Irish sample. As a sensitivity analysis, an ANOVA was conducted to investigate the effect of ethnicity on body image scores, the tests revealed no significant effect of ethnicity on body image scores (F(5, 80) = 0.645, *p* = 0.666, η^2^_p_ = 0.039) indicating that ethnicity is not a covariate in the analysis.

To determine the appropriate sample size for the regression models, an a priori power analysis was conducted using G*Power [82]. The analysis considered the most complex model used, which consisted of 4 predictors and a medium effect size (f2 = 0.15), with an alpha level of 0.05 and a power of 0.80. Based on this, it was determined that a sample size of N = 85 would be suitable for the regression model.

### 2.2. Questionnaires

#### 2.2.1. Body Image Disturbance

To create a multidimensional measure of BID and explore its connection to interoception and the body schema in both men and women, we utilized the conceptualization of BID described in Naraindas and Cooney (2023) [53]. For a detailed theoretical justification, please refer to the referenced paper.

##### Trait Body Dissatisfaction

This was measured by the body shape questionnaire (BSQ) [83] The BSQ version used in the study involves 30 questions (four questions were omitted as they were about weight control/eating disorder behaviours and were not necessary for our study). Although it is typically used to investigate shape concerns in female populations [84,85], the male version of the scale has been validated to detect shape concerns occurring in men [86,87]. In this study, men received the male version of the BSQ, and women received the female version.

##### State Body Dissatisfaction

The body image states scale (BISS) [30] is a 6-item questionnaire that was used to measure participants’ evaluation and affect towards their physical appearance at that moment using a 9-point Likert scale, with a higher score indicating a higher body dissatisfaction. Although the scale has been typically used in females [88], other studies have found the regular version of the scale to be valid for males [89].

##### Objectified Body Consciousness

Body shame and body surveillance were measured in this study using their subsequent subscales from the objectified body consciousness questionnaire (OBC). These subscales contain 8 questions each, scored on a 7-point Likert scale from “strongly agree” to “strongly disagree”. Both the subscales have been shown to be valid in male populations [90].

#### 2.2.2. Interoceptive Sensibility 

This was measured by the multidimensional assessment of interoceptive awareness, version 2 (MAIA-2) [55]. The MAIA-2 consists of 37 questions, with responses rated on a 5-point Likert scale ranging from “Never” to “Always”. This scale has been used to assess interoceptive sensibility in both female and male populations [57]. The MAIA-2 is divided into 8 sub-scales including: Noticing, Not-distracting, Not-Worrying, Attention Regulation, Emotional Awareness, Self-Regulation, Body Listening, and Trusting [55]. For each of the eight subscales, the score was calculated by averaging the scores of items belonging to the subscale [55]. We also created a total MAIA-2 score by computing the mean of the scores of the sub-scales as an index of overall interoceptive sensibility as done in prior studies [91,92,93]. While the utilization of a total score is not usually advised for the MAIA-2 [55], we sought to capture an overall index of IS to include in a regression model. Higher scores, both overall and on individual subscales, are indicative of higher internal body awareness. This scale has been used to assess IS in both men and women [57].

#### 2.2.3. Own Body Transformation Task (OBT)

**Figure 1:**
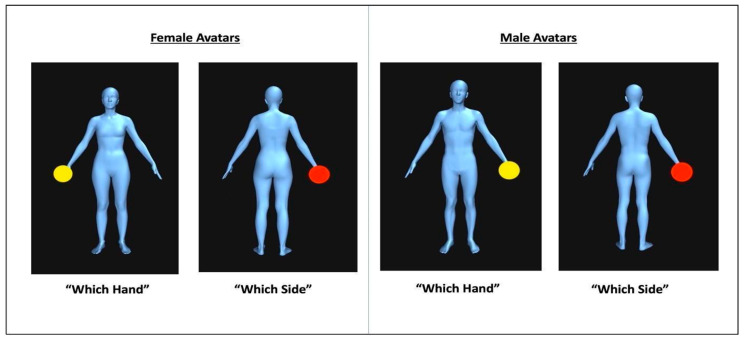
Example of the male and female versions of the avatars from the OBT task displayed in the “which hand” condition as denoted by the red ball (experimental) and the “which side” condition as denoted by the yellow ball (control).

The OBT task employed in this study incorporates the same adaptations and considerations as described in Naraindas and Cooney (2023) [53]. In the OBT task, participants are asked to make laterality judgements regarding the handedness of a marked hand of an avatar presented in different positions. Typically, reaction times and error rates increase with increased disparity between the participant’s own body position and the avatar’s position, reflecting the cost of making an embodied transformation. The stimuli consisted of full-body avatars in front-facing, side-facing, and back-facing positions. One modification in this study involved male participants viewing male dimorphic avatars, while female participants viewed female dimorphic avatars (see Figure 1). These avatars were depicted holding either a red or yellow ball in their left or right hand (See Figure 1) and were presented in various angles (0 and 90 degrees) and the body of the avatars was made to appear marginally over and under the average BMI weight (by ±5 BMI points). The avatars were made on https://bodyvisualizer.com/, accessed on 19 January 2022 [94] and PsychoPy [77] was used to present the stimuli.

In the experimental trials, participants were instructed to imagine the avatar body as their own and to decide on which hand the yellow ball was placed. Participants were instructed to respond quickly but accurately by pressing the left or right arrow key, to determine on what hand the circle was placed. Control trials featured a red ball instead of a yellow ball and were interspersed within the blocks, where participants were instructed to determine the side of the screen (not the hand) where the ball was located. The time taken to make judgments of “which side” was to verify that the response time was due to mental transformations as opposed to left-right judgment [95].

### 2.3. Procedure

Following Naraindas and Cooney (2023) [53], the male data were collected online, with participants instructed to perform the tasks on a laptop or computer. The order of the questionnaires and OBT tasks was counterbalanced.

During the OBT task, participants received instructions on how to perform the tasks and then completed 10 practice trials. In the experimental phase, participants were given four blocks, each consisting of 36 trials. Each block included 24 experimental trials and 12 control trials, resulting in a total of 96 experimental trials. The experimental trials encompassed two orientations (0 degrees and 90 degrees), three positions (front-facing, back-facing, and side-facing), and two weight conditions (underweight/overweight). Each unique combination of stimuli was presented once in each block. After completing the tasks, participants were directed to the questionnaire portion of the study where they were given the questionnaires assessing BID and IS. We used effect size measures (Cohen’s d for *t*-tests and partial eta-squared for ANOVA), and applied Greenhouse–Geisser corrections when Mauchly’s sphericity test was significant. Bonferroni-corrected post hoc comparisons were performed in cases of statistically significant main effects and interactions for ANOVA results.

## 3. Data Analysis

First, we calculated descriptive statistics for all behavioural measures in this study. The BID composite score was made up of the four questionnaires (the BSQ, BISS, and the body shame and body surveillance subscales of the OBC) following Naraindas and Cooney (2023) [53]. Summary statistics are provided for all behavioural measures by gender in Table 1.

Due to incomplete data sets, there were only 75 complete men’s data files compared to 86 complete women’s data files for the OBT task. Furthermore, due to high levels of inaccuracies of side-facing trials in female participants (50.77%), they were dropped from the analysis as they could not be directly compared to male trials. Response latencies faster than 200 ms. and slower than 5000 ms. (0.8% of trials) were removed. Statistical analyses were conducted using R Statistics [96] and JASP [97].

## 4. Results

### 4.1. Gender Differences in BID and IS

The data confirmed the assumptions of parametric statistical analyses, as such a series of one-way analysis of variance (ANOVA) analyses were conducted to investigate gender differences in overall body image disturbance and to determine differences in each behavioural measure as reported in Table 1. The ANOVA results indicated significantly higher BID composite scores in women compared to men (F(1, 170) = 21.57, *p* < 0.001, η^2^_p_ = 0.113).

**Figure 2:**
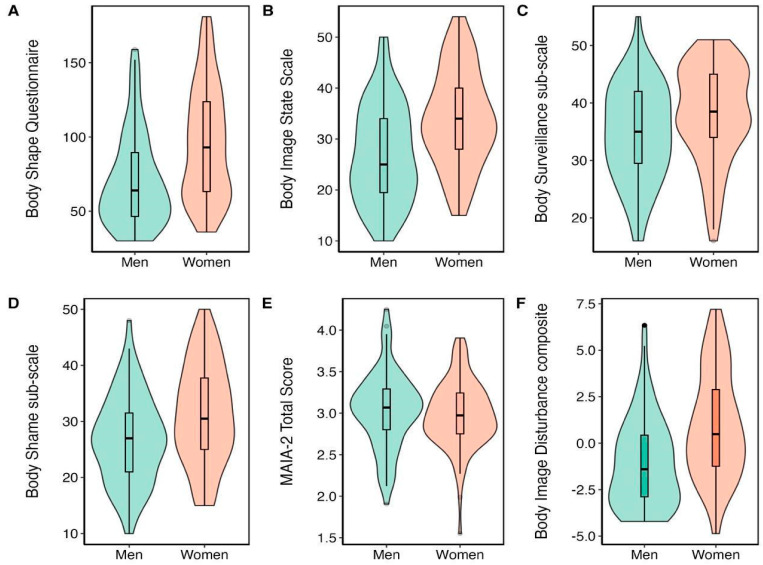
Mean body image and MAIA-2 scores demonstrating gender differences in (**A**): body image state scale, (**B**): body shape questionnaire, (**C**): body shame sub-scale, (**D**): body surveillance sub-scale and (**E**): MAIA-2 total score and (**F**): body image disturbance composite score (Bold).

Additionally, three one-way ANOVAs were conducted on the body image scales (see Table 1 for means and standard deviations), the results indicated that women reported significantly higher scores than men: in the body shape questionnaire (F(1, 170) = 22.79, *p* < 0.001, η^2^_p_ = 0.118), the body image state scale (F(1, 170) = 21.38, *p* < 0.001, η^2^_p_ = 0.112), The body shame subscale (F(1, 170) = 8.778, *p* < 0.05, η^2^_p_ = 0.049) and the body-surveillance subscale (F(1, 170) = 4.412, *p* < 0.05, η^2^_p_ = 0.025), indicating higher levels of BID overall. Figure 2 illustrates the gender differences in body image and MAIA-2 total scores.

A one-way ANOVA was conducted to investigate gender differences in the MAIA-2. The results demonstrated no significant gender difference in MAIA-2 total scores (F(1, 170) = 0.722, *p* = 0.397, η^2^_p_ = 0.005) (See Table 1). However, on the sub-scales, women reported significantly higher scores on the Not Distracting and Not Worrying scales, whereas men reported significantly higher scores on the Attention Regulation, Body Listening, and Body Trusting scales (See Table 1).

### 4.2. Does Gender and Interoceptive Sensibility Predict Body Image Disturbance?

**Figure 3:**
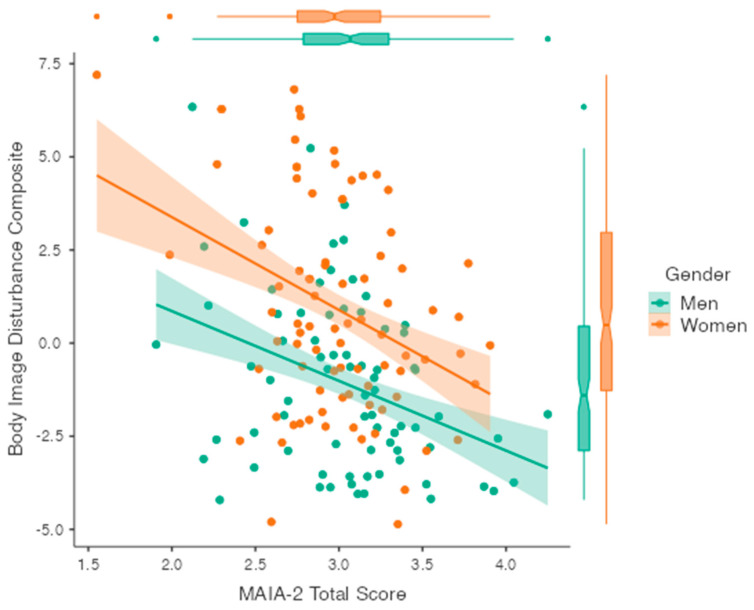
Scatter plot showing composite body image disturbance scores on the y-axis and interoceptive sensibility on the x-axis with separate regression lines plotted for men and women. Boxplots for MAIA-2 and body image disturbance composite scores are displayed on the sides of the plot.

A hierarchical regression was conducted using the BID composite score as the dependent variable and gender and IS total score as predictors. Gender was dummy-coded and entered the null model, with men as the reference group to account for gender-related variance. The first step was significant (R^2^ = 0.128, F(1, 159) = 23.411, *p* < 0.001) and Gender explained 12.8% of the variance in BID. After the entry of IS to the second step of the model, the total variance explained by the model was 23.5% (R^2^ = 0.235, F(1, 158) = 24.302, *p* < 0.001). The introduction of IS explained an additional 10.7% of the variance in BID after accounting for gender. The final adjusted model demonstrated that gender (men) emerged as a significant negative predictor of BID (SE = 0.394, t = −4.822, *p* < 0.001). IS also emerged as a negative predictor of BID (SE = 0.460, t = −4.700, *p* < 0.001) (see Figure 3). Exploratory Pearson’s partial correlations were conducted to investigate the relationship of the MAIA-2 sub-scales with all body image scales after factoring for gender (see Figure S1).

### 4.3. Gender Differences in the OBT Task

#### 4.3.1. Performance

Overall, errors were made on 4.5% of all trials, 1.11% for women, and 7.71% for men, indicating an above-chance performance. Women demonstrated overall higher accuracy (M = 98.88, SD = 22.25) than men (M = 92.29, SD = 15.80) in the task. Overall, the mean reaction time in the task was 1410 ms; 1472 ms (SD = 547.20) for women and 1353 ms (SD = 543.25) for men.

#### 4.3.2. Experimental vs. Control Condition

To examine the efficacy of the experimental versus control conditions we ran a 2 × 2 ANOVA with *condition* (control, experimental) and *position* (front-facing, back-facing) as within subjects’ factors and accuracy (% correct) as the dependent variable. Only the 0-degree experimental trials were included, as control trials were only presented in 0 degrees. The analysis revealed a main effect of condition (F(1, 662) = 6.177, *p* = 0.013, η^2^_p_ = 0.009) and Position (F(1, 662) = 20.106, *p* < 0.001, η^2^_p_ = 0.029) on Accuracy. There was no significant position × condition interaction on accuracy (F(1, 662) = 0.665, *p* = 0.415, η^2^_p_ = 0.001). Post hoc tests indicated that participants’ performance was significantly more accurate for control trials (M = 95.46, SD = 12.60) than for experimental trials (M = 92.89, SD = 14.32) (t = 2.485, SE = 1.03, *p* = 0.013, d = 0.193) in all positions except for the back-facing position where participants yielded similar accuracy across both conditions (for full post-hocs, see Table S1 in Supplementary Materials).

#### 4.3.3. Egocentric Transformation

To analyse the data, we calculated the mean RT on correct trials for the orientation, weight, and posture of the avatar across genders. Subsequently, we conducted a 2 × 2 × 2 × 2 omnibus mixed-model ANOVA, with position (front-facing, back-facing), orientation (0 degrees, 90 degrees), and weight (underweight, overweight) as within-subject factors, and gender (men, women) as the between-subjects factor (see Table S2 for full ANOVA results). The ANOVA revealed a significant main effect of position (F(1, 167) = 196.2, *p* < 0.001, η^2^_p_ = 0.540), with back-facing trials having significantly quicker RTs (M = 1194.55 ms, SD = 450.89) than front-facing trials (M = 1627.21 ms, SD = 722.30). There was also a significant interaction between position and orientation (F(1, 167) = 27.04, *p* < 0.001, η^2^_p_ = 0.139), with there being significant differences between positions presented in 0 and 90 degrees (see Table S3 for fully reported post-hocs). There were no main effects of orientation or weight and no between subjects’ effects of gender on reaction time.

Based on the results of the ANOVA, participants displayed an egocentric transformation effect. To explore this effect further, we computed an egocentric transformation index by subtracting the mean RT for the avatar in the front-facing condition from the back-facing condition (see Table 2). Since avatar weight did not have a significant impact on reaction times, it was excluded from further analysis.

A two-way ANOVA was conducted to investigate differences in egocentric transformation cost between gender and orientation. The ANOVA revealed a main effect of orientation (F(1, 316) = 17.915, *p* < 0.001, η^2^_p_ = 0.054) on egocentric transformation cost. Post-hoc tests revealed that the 0-degree condition yielded a larger transformation cost (M = 5500 ms, SD = 6220) than the 90-degree condition (M = 3220 ms, SD = 4940) (t = 4.233, SE = 0.053, *p* < 0.001, d = 0.475). There were no significant main effects of gender (F(1, 160) = <0.001, *p* = 0.957, η^2^ < 0.001)) and no significant gender*orientation interaction on the egocentric transformation cost (F(1, 316) = 2.051, *p* = 0.153, η^2^_p_ = 0.006). However, exploratory *t*-tests revealed that there was a significant difference in the egocentric transformation cost between the 0- and 90-degree conditions in women (t = 4.165, SE = 0.072, *p* < 0.001, d = 0.635) but not men (t = 1.910, SE = 0.078, *p* = 0.342, d = 0.314) (see Table 2 for means and standard deviations).

### 4.4. Does BID and IS Predict Egocentric Transformation Cost?

A hierarchical regression analysis was conducted to look at the effect of body image disturbance on OBT task performance. The egocentric transformation cost was entered as the outcome and gender, the BID composite, IS, and orientation were entered as predictors in line with Naraindas and Cooney (2023) [53]. Gender and orientation were entered into the null model to partially out their independent variance on the egocentric transformation cost. The first step was significant (R^2^ = 0.056, F(2, 319) = 9.490, *p* < 0.001) and gender and orientation explained 0.56% of the variance in the egocentric transformation cost. After the entry of BID and IS to the second step of the model, the total variance explained by the model was 0.63%. The introduction of BID and IS did not lead to a significant change over the null model (*p* = 0.335) but still contributed to a significant model (F(4, 319) = 5.298, *p* < 0.001). However, only orientation (90 degrees) emerged as a significant negative predictor of the egocentric transformation cost (SE = 0.053, t = −4.356, *p* < 0.001). See Supplementary Table S4 for fully reported standard errors and coefficients for all variables.

## 5. Discussion

The present study examined gender differences in key features of body image disturbance (body objectification, state, and trait body dissatisfaction) and interoceptive sensibility. We also wanted to understand how BID and IS interact with the body schema—as measured by a motor imagery task, and if this differed between men and women.

Consistent with our first hypothesis, women demonstrated significantly higher levels of BID across all scales and the composite score. This finding is in line with prior research indicating that women consistently demonstrate a higher prevalence of negative body image [98,99,100] than men. Increased BID in women can be explained by sociocultural theory which posits that the social environment (e.g., media, peer dynamics, parental expectations, and relationships) exerts substantial pressures on women to conform to prevailing body ideals and regard their bodies negatively [101,102]. Even though there seems to be increasing cultural emphasis on male body ideals [103], appearance focus remains more central to the concept of feminine gender roles [104]. Indeed, men report lower body concerns and pressures to conform to idealised body standards compared to women [27,105]. This can also be ascribed to factors related to gender socialisation and body talk, wherein men frequently encounter increased expectations for promoting positive and self-accepting discussions about their bodies than women [106,107]. For example, in a study conducted by Voges et al. (2019), participants were presented with images featuring various body types, with their own head or another person’s head imposed on these bodies [24]. The study found that men assign higher ratings to bodies featuring their own heads, whereas women tend to be more self-critical and are less influenced by identity when rating bodies [24]. This indicates that men are likely to make more favourable judgements towards their bodies compared to women.

One explanation for this is that masculine stereotypes typically emphasise traits like pride, dominance, and success which encourage men to evaluate their bodies favourably when compared to body ideals, enabling self-promotion [108]. For women, it is normalised to participate in more critical conversations about their body shapes (e.g., fat talk) and body dissatisfaction amongst other women [109,110]. Women experience greater pressure to assess their bodies in a self-deprecating manner to appear agreeable and emphasise commonalities [111]. Indeed, our study found that women reported higher weight and shape concerns (in the BSQ) and evaluated their bodies more negatively in the present moment (in the BISS) compared to men. An accumulating body of research indicates that body talk and fat talk lead to body image disturbance across several domains e.g., body dissatisfaction, body surveillance, and body shape concerns [112]. Therefore, the higher BISS and BSQ scores in women in our study could be explained by women’s social pressures to assess their bodies more critically and with more scrutiny than men.

Moreover, our study also found elevated body surveillance and shame scores among women, indicating a stronger tendency towards self-objectification compared to men. According to objectification theory [37], body surveillance results in body shame as it prompts women to assess their bodies against unattainable internalised body ideals [36]. As such, self-objectification in women tends to be more pronounced among younger women, particularly in college-aged women (aged 18–25) [113]. This is due to increased social media use and image sharing [114], the formation of new peer networks [115], and increased appearance investment [116] typically seen in this age group. Therefore, the higher body surveillance and shame scores observed in women in this study may be indicative of an age cohort often associated with a greater inclination toward self-objectification.

Furthermore, although high levels of self-objectification are commonly observed among young women [117], it manifests differently across different subgroups of men who may be selectively vulnerable to these concerns [39,40]. This form of objectification exhibits distinct features, including a greater emphasis on muscularity rather than thinness as a prevailing societal standard of attractiveness [38]. For example, Hallsworth, Wade, and Tiggeman (2005) found that male bodybuilders report higher levels of self-objectification than controls and that this was related to an increased drive for muscularity [118]. Furthermore, homosexual men demonstrate significantly higher levels of self-objectification than heterosexual men [119] and in some studies, even heterosexual women [120]. Additionally, it’s crucial to acknowledge that transgender and non-binary individuals have distinct body image experiences compared to cisgender individuals, resulting from their unique experiences with minority stress and gender dysphoria [121]. Research indicates that both transwomen and transmen report elevated levels of body dissatisfaction and body surveillance compared to cisgender controls, and this is associated with their desire to achieve a more gender-affirmative appearance [122]. As we did not account for gender-variant groups in the current study, it is unclear whether the gender differences may be generalised to a more representative sample. Future research should investigate gender-variant populations to identify the unique vulnerabilities that may contribute to different aspects of BIDs.

Regarding gender differences in the MAIA-2, there were no significant differences in total scores. However, we found that women had significantly higher scores in the Not-Distracting, Not-Worrying sub-scales, indicating that they were less likely to experience excessive concern towards their bodily sensations and were less inclined to use distracting behaviours to numb or avoid their bodily sensations. Alternatively, men scored higher than women on Attention Regulation, Body Trusting, and Body Listening sub-scales. This indicates that men exhibit a greater capacity to regulate their attention toward bodily sensations and display confidence in trusting and attending to these sensations. These findings are consistent with prior research that has explored gender differences in IS, albeit to a certain degree. For instance, similar to our study, Todd et al. (2019) also observed significantly higher attentional regulation and body trusting scores in men compared to women [57]. However, contrary to our results, Grabauskaite et al. (2017) found that women scored lower on Not-distracting and Not-worrying compared to men [45]. It is important to note that the Not-Distracting and Not-Worrying sub-scales have the lowest internal consistency, which could reflect the contradictory results across different studies [55,57,123].

Nevertheless, the gender differences in IS noted in this study can also be explained by the significant disparities in reproductive health between men and women. Dynamic changes in women’s reproductive physiology (e.g., puberty, menstruation, pregnancy) contribute to a noisier body environment, reducing a woman’s likelihood of excessive concern and worry about bodily sensations compared to men [124] as reflected in the responses to the Not-Worrying and Not-Distracting sub-scales. Additionally, societal normalisation of the adverse and unfavourable aspects of menstruation often causes women to downplay and be sceptical of their internal sensations generally [125]. This might explain the reduced inclination among women to demonstrate excessive attention and trust toward bodily sensations when compared to men as seen in their responses to the Body Trusting, Listening, and Attention Regulation sub-scales.

An important finding of the study was the gender-invariant negative relationship between IS and BID. While gender had the most substantial contribution to BID, it’s noteworthy that interoceptive sensibility made a similarly significant unique negative contribution to BID. This indicates that both women and men with higher BID also demonstrated lower levels of interoceptive sensibility. This result extends the findings of Todd et al. (2019) who found that after accounting for gender, negative body image (overweight preoccupation and appearance preoccupation) was predicted by MAIA-2 scores [57]. As such, the findings of our study support the generalizability of the ALT across genders, suggesting that both men and women with BIDs may prioritize external signals and consequently exhibit lower levels of internal awareness [75].

The associations between IS and various aspects of body image, as well as their importance in understanding the development of eating disorders, have mainly been studied in women with limited investigation in men. For example, Peat et al. (2011) found that IS was a significant mediator in the relationship between self-objectification and eating disorder symptoms in women [126]. A recent longitudinal study by Grunewald et al. (2023) showed that conformity to masculine norms predicted IS, which then predicted muscle dysmorphic symptoms in men [62]. While decreased interoceptive ability has typically been seen as a pathway to restrictive eating behaviours in women [127,128], our study indicates that this association remains consistent across genders, highlighting its potential role in contributing to eating pathology in men. For instance, diminished awareness of internal sensations could result in men overeating during bulking phases in their pursuit of muscle gain, ignoring their hunger cues during cutting phases, and persisting in demanding weight training despite discomfort [20,129]. Therefore, future research must explore the relationships between IS and body image concerns specific to men (e.g., drive for muscularity) to determine if these relationships have similar implications for eating pathology.

Regarding performance in the OBT task, all participants, regardless of gender, displayed faster rotation times when dealing with the back-facing avatar, consistent with the typical findings in the OBT task [130]. However, we found no significant gender differences in the egocentric transformation cost, indicating that both men and women took comparable amounts of time to rotate a front-facing avatar from a back-facing one. Although gender differences in mental rotation ability usually favour men over women [74], these differences are known to be less pronounced with stimuli depicting entire human bodies [131,132]. Despite the relationship between interoception, gender, and body image, we did not find a significant contribution of IS, gender, and BID on motor imagery performance (the egocentric transformation cost). These findings contradict our previous study which demonstrated a relationship between BID and body schema across different stages of female adulthood [53]. As the current study included the unique variance of a male sample, the results indicate that BID in men may not influence the body schema (as assessed by motor imagery) in a similar manner to women. This potentially limits the applicability of the ALT, indicating that BID may not be related to alterations in the body schema (i.e., egocentric processing) in men.

However, performing an egocentric mental transformation in the OBT task depends on the degree of embodiment, affecting the extent to which one engages various components of their body schema during mental self-rotation [133]. Research indicates that women employ more embodied strategies, actively involving their egocentric reference frames in spatial perspective tasks (mapping their mental representation of their body to the avatar’s perspective) [134,135]. In contrast, men often adopt a disembodied rule-based approach, such as (“their left is my right”) when making spatial perspective judgements [134,135]. Therefore, men may trade empathetic depth for higher speed when compared to women, due to employing more “systematising” approaches [133]. Indeed, in the current study, men demonstrated overall faster performance when responding to both front-facing and back-facing avatars, indicating a preference for speed. Conversely, women displayed overall higher accuracy than men, highlighting their emphasis on accurate embodied spatial perspective-taking.

Additionally, whilst no significant gender differences were observed in overall egocentric transformation cost, we found that women had significantly higher egocentric transformation costs at 0 degrees than at 90 degrees whereas men performed similarly across orientations. Anatomically familiar orientations (0 degrees) are typically processed faster than unfamiliar orientations (90 degrees) as they are more embodied [136]. Therefore, larger egocentric transformation costs in 0 degrees in women, might suggest a tendency for women to employ more egocentric strategies in the task compared to men. Therefore, the OBT task performance observed in the study may have been influenced by gender-specific strategies and not by BID.

## 6. Limitations and Future Directions

Regarding gender differences in body image, we may be observing cohort effects due to the cross-sectional nature of the study [137]. Young adult women are typically noted to display higher levels of negative body image than at any other time in their lifespan [138], whilst younger men report experiencing lower levels of negative body image in young adulthood than at other times in their lifespan [139]. A 20-year longitudinal study by Keel et al. (2007) found that as men aged, they showed increasing weight and body dissatisfaction, while women—despite similar weight increases—experienced stable levels of negative body image across adulthood [140]. Therefore, although younger men report overall lower levels of BID in this study, different components of BID may peak at different times across the lifespan between genders.

Overall, this study shows that, despite the generally higher levels of BID among women compared to men, the relationships between body image and interoceptive sensibility remain consistent, independent of gender. However, the present study relied on a self-report explicit measure of interoception (the MAIA-2 questionnaire). Interoception varies across body systems (e.g., gastric, respiratory, etc.), domains (explicit, implicit), and assessment methods (e.g., self-report, experiments, behavioural tasks) [141,142]. Therefore, it is advisable for future research to explore whether the association between BID and IS can be confirmed across various measures of interoceptive propensity.

Overall, the findings of the study hold significant implications. Given that altered interoceptive processing is linked to BIDs, and this is considered a risk factor for developing EDs in women [143], our findings suggest that these comparable relationships exist across genders, highlighting a vulnerability in men as well. This highlights the importance of incorporating interoceptive enhancement techniques into body image improvement programs and engaging men within these programmes. Future research should explore male-specific facets of body image and its relationship to other dimensions of interoception, and the body schema.

## Figures and Tables

**Table 1:** Means, standard deviations (in brackets), and results of the ANOVAs for the gender differences for body image and interoceptive sensibility variables.

Questionnaires	Women (N = 85) M (SD)	Men (N = 85) M (SD)	F	*p*	η^2^_p_
Body image disturbance composite	0.937 (2.870)	−0.937 (2.402)	21.57	<0.001	0.113
BSQ	95.60 (37.67)	70.61 (30.57)	22.79	<0.001	0.118
BISS	33.97 (9.43)	27.26 (9.60)	21.38	<0.001	0.112
Body shame sub-scale	31.35 (8.84)	27.58 (7.80)	8.79	0.003	0.049
Body-surveillance sub-scale	38.10 (8.33)	35.44 (8.29)	4.41	0.037	0.025
MAIA-2 total	2.98 (0.40)	3.04 (0.45)	0.72	0.397	0.005
MAIA-2: Noticing	3.45 (0.67)	3.40 (0.75)	0.19	0.663	0.001
MAIA-2: Not Distracting	2.96 (0.68)	2.70 (0.76)	5.63	0.019	0.032
MAIA-2: Not Worrying	2.78 (0.53)	2.17 (0.92)	26.83	<0.001	0.146
MAIA-2: Attention-Regulation	2.92 (0.60)	3.17 (0.64)	6.42	0.012	0.037
MAIA-2: Emotional Awareness	3.38 (0.78)	3.53 (0.76)	1.66	0.200	0.010
MAIA-2: Self-Regulation	2.78 (0.73)	2.98 (0.85)	2.81	0.096	0.016
MAIA-2: Body Listening	2.61 (0.76)	2.92 (0.79)	6.59	0.011	0.038
MAIA-2: Trusting	2.94 (0.86)	3.46 (0.88)	15.11	<0.001	0.082

**Table 2:** Mean reaction times and standard deviations (in brackets) in milliseconds on correct trials of the OBT task indicate the performance of men and women across different orientations, positions, and egocentric transformations.

Gender	Back-Facing		Front-Facing		Egocentric Transformation Cost [Frontfacing-Backfacing]	
	0 Degrees	90 Degrees	0 Degrees	90 Degrees	0 Degrees	90 Degrees
Men (N = 74)	1155 (354)	1265 (357)	1663 (596)	1625 (549)	5090 (455)	3600 (459)
Women (N = 86)	1156 (430)	1336 (501)	1741 (843)	1621 (604)	5850 (592)	2850 (349)

## Data Availability

The data presented in this study are openly available at https://osf.io/24e98/?vie, accessed on 23 November 2023.

## Supplementary Materials

The following supporting information can be downloaded at: https://www.mdpi.com/article/10.3390/bs14010025/s1, Figure S1: Pearsons’ partial correlation heat-map of body image disturbance measures and interoceptive sensibility measures (conditioned on gender); Table S1: Post Hoc tests for interactions between position and condition on accuracy for ANOVA 4.3.2; Table S2: Mixed model ANOVA results for reaction time analysis; Table S3: Post Hoc Comparisons for Position x Orientation interaction for ANOVA 4.3.3; Table S4: Regression results for the effect of BID and IS on the egocentric transformation cost.
