# Supplementary material for: Beyond Gender: Interoceptive Sensibility as a Key Predictor of Body Image Disturbances
Source: Behav Sci (Basel). 2023 Dec 28;14(1):25. doi: 10.3390/bs14010025 (PMC10812832; doi:10.3390/bs14010025)
Supplement: Supplementary file 1 [file behavsci-14-00025-s001.zip › behavsci-2763265-supplementary.pdf]

Supplementary Tables and Figures

Figure S1. Pearson's Partial Correlation heat-map of Body Image disturbance measures and interoceptive sensibility measures (conditioned on gender): Purple indicates positive associations and brown indicates negative associations. Significance as follows: * $p < 0.05$, ** $p < 0.01$, *** $p < 0.001$

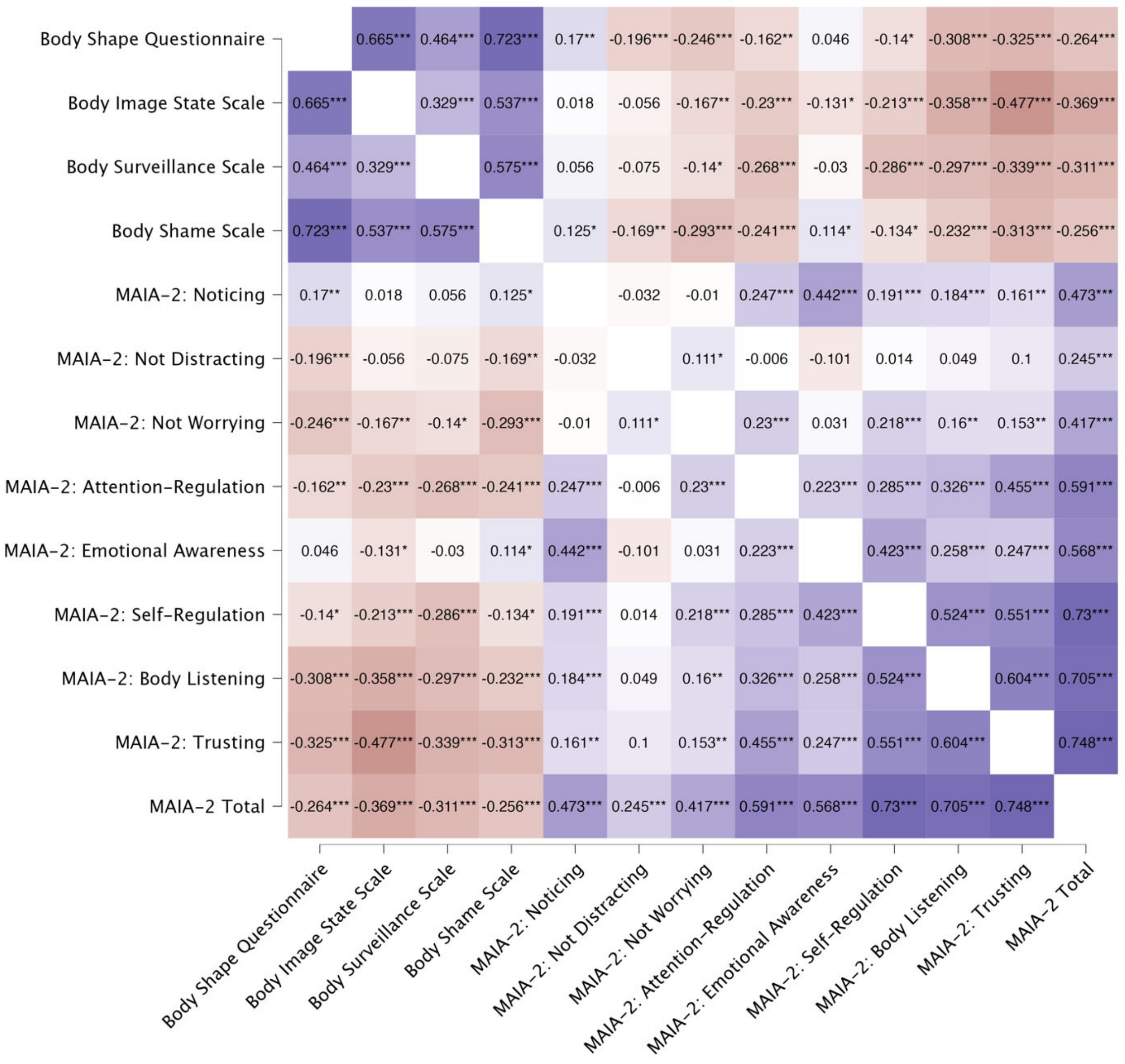

Table S1. Post Hoc tests for interactions between position and condition on accuracy for ANOVA 4.3.2

		Mean Difference	SE	t	Cohen's d	p_{bonf}
BACKFACING Control	FRONTFACING Control	3.782	1.460	2.590	0.284	0.059
	BACKFACING Experimental	1.722	1.456	1.182	0.129	1.000
	FRONTFACING Experimental	7.186	1.456	4.936	0.540	< .001
FRONTFACING Control	BACKFACING Experimental	-2.061	1.460	-1.411	-0.155	0.952
	FRONTFACING Experimental	3.403	1.460	2.331	0.256	0.120
BACKFACING Experimental	FRONTFACING Experimental	5.464	1.456	3.753	0.411	0.001

Note. P-value adjusted for comparing a family of 4

Table S2. Mixed model ANOVA results for reaction time analysis

Cases	Sum of Squares	df	Mean Square	F	p	η^2_p
Position	64.412	1	64.412	196.200	< .001	0.540
Position ✻ gender	0.142	1	0.142	0.431	0.512	0.003
Residuals	54.825	167	0.328			
Orientation	0.153	1	0.153	1.136	0.288	0.007
Orientation ✻ gender	0.039	1	0.039	0.290	0.591	0.002
Residuals	22.434	167	0.134			
Weight	0.052	1	0.052	0.390	0.533	0.002
Weight ✻ gender	0.268	1	0.268	2.012	0.158	0.012
Residuals	22.206	167	0.133			
Position ✻ Orientation	4.471	1	4.471	27.036	< .001	0.139
Position ✻ Orientation ✻ gender	0.400	1	0.400	2.419	0.122	0.014
Residuals	27.619	167	0.165			
Position ✻ Weight	6.786×10^{-4}	1	6.786×10^{-4}	0.005	0.944	2.961×10^{-5}
Position ✻ Weight ✻ gender	3.064×10^{-4}	1	3.064×10^{-4}	0.002	0.962	1.337×10^{-5}
Residuals	22.919	167	0.137			
Orientation ✻ Weight	0.058	1	0.058	0.435	0.511	0.003
Orientation ✻ Weight ✻ gender	0.350	1	0.350	2.623	0.107	0.015
Residuals	22.299	167	0.134			
Position ✻ Orientation ✻ Weight	1.803×10^{-4}	1	1.803×10^{-4}	0.001	0.970	8.395×10^{-6}
Position ✻ Orientation ✻ Weight ✻ gender	0.566	1	0.566	4.403	0.037	0.026
Residuals	21.477	167	0.129			

Note. Type III Sum of Squares

Table S3. Post Hoc Comparisons for Position ∗ Orientation interaction for ANOVA 4.3.3

		Mean Difference	SE	t	Cohen's d	p_{bonf}
Back-facing, 0	Front-facing, 0	-0.552	0.038	-14.432	-0.843	< .001 ***
	Back-facing, 90	-0.136	0.030	-4.576	-0.208	< .001 ***
	Front-facing, 90	-0.458	0.037	-12.374	-0.699	< .001 ***
Front-facing, 0	Back-facing, 90	0.416	0.037	11.225	0.634	< .001 ***
	Front-facing, 90	0.094	0.030	3.149	0.143	0.011 *
Back-facing, 90	Front-facing, 90	-0.322	0.038	-8.413	-0.491	< .001 ***

* p < .05, ** p < .01, *** p < .001

Table S4. Regression results for the effect of BID and IS on the egocentric transformation cost

Coefficients

**Model**		**Unstandardized**	**Standard Error**	**Standardized[a]**	**t**	**p**
H_0	(Intercept)	0.548	0.044		12.433	< .001
	Orientation (90)	-0.227	0.052		-4.355	< .001
	Gender (Male)	0.007	0.052		0.132	0.895
H_1	(Intercept)	0.828	0.203		4.080	< .001
	MAIA-2 Total	-0.012	0.008	-0.083	-1.432	0.153
	Body Image Disturbance Composite	-0.002	0.011	-0.009	-0.143	0.886
	Orientation (90)	-0.227	0.052		-4.356	< .001
	Gender (Male)	0.009	0.056		0.164	0.869

[a] Standardized coefficients can only be computed for continuous predictors.
